# Animal Social Network Structures Across the Fast‐Slow Continuum

**DOI:** 10.1111/ele.70449

**Published:** 2026-08-23

**Authors:** Ross S. Walker, Andy White, Xander O'Neill, Nina H. Fefferman, Matthew J. Silk

**Affiliations:** ^1^ Maxwell Institute for Mathematical Sciences and Department of Mathematics Heriot‐Watt University Edinburgh UK; ^2^ Institute of Ecology and Evolution University of Edinburgh Edinburgh UK; ^3^ Department of Mathematics University of Tennessee Knoxville USA; ^4^ Department of Ecology and Evolutionary Biology University of Tennessee Knoxville USA; ^5^ National Institute for Modeling Biological Systems (NIMBioS) Knoxville USA

**Keywords:** homophily, preferential behaviour, self‐organisation, social dynamics, social inheritance, social networks, speed of life

## Abstract

The fast‐slow continuum is a key axis of variation in life history strategies capturing the evolutionary trade‐off between allocation in lifespan versus reproduction. It has been suggested that social behaviour, and therefore network structure, may vary across this continuum, but formal theory remains scarce. We develop a mathematical model to examine how the rate of demographic replacement may influence emergent social network structures under a range of social preferences and modes of social inheritance. Our key finding is that rapid demographic replacement can constrain emergent social network structure. We predict that generation time determines the drivers of variation in network structures; for longer generation times, emergent network properties are primarily influenced by social preferences, whereas for shorter generation times it is social inheritance. Variation in generation time alone can lead to substantial shifts in network properties; therefore, diversity in life history strategies can help explain diversity in social network structures.

## Graphical abstract

Using a mathematical model, we examine how the rate of demographic replacement (represented by generation time) influences emergent social network structure in natural populations. By considering a range of social preferences and modes of social inheritance, we find that demographic replacement influences the extent to which social networks can self‐organise. Generation time determines whether network structure is primarily shaped by social preferences or by social inheritance.

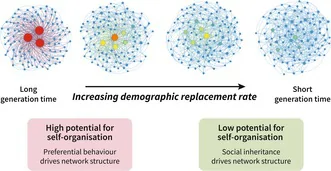

## 1 Introduction

Life history strategies are defined by trade‐offs between survival, development and behaviour, which are in turn subject to constraints imposed by resource availability, environmental conditions and allometry (Stearns 1998; Healy et al. 2019). A well‐known trade‐off that shapes life history strategies is the *fast‐slow continuum*, describing the relative allocation in lifespan and reproduction, termed *speed of life* (Silk and Hodgson 2021; Healy et al. 2019). Species with a slow speed of life are characterised by long lifespans but low reproductive rates (i.e., long generation times). Such species often have greater levels of maternal care and improved juvenile survival rates, e.g., killer whales ( *Orcinus orca* ) or African elephants ( *Loxodonta africana* ) (Zipple et al. 2024; Weiss et al. 2023; Hoerner et al. 2023). Conversely, species with a fast speed of life have a high reproductive rate but short lifespan (i.e., short generation times), often exhibiting higher metabolic rates than slow‐lived species, postulated to sometimes have behavioural consequences under the pace of life syndrome hypothesis (Réale et al. 2010; Biro and Stamps 2010; Polverino et al. 2018, although see Royauté et al. 2018). As the fast‐slow continuum is a key axis of variation in life history strategies, it is important to understand how it is associated with other population characteristics, such as social structure. However, few studies have explicitly tested how social behaviour varies across the fast‐slow continuum (Salguero‐Gómez 2024).

Individual social relationships collectively define the social network structure of a group or population (Krause et al. 2014; Sumpter 2005), and this structure can be shaped over time by demographic processes such as replacement (the natural birth and death of individuals) (Shizuka and Johnson 2020). The impact of demographic replacement can be both direct, through removal and addition of possible social partners, and indirect, inducing behavioural changes that shape social dynamics that then modify network structure (Shizuka and Johnson 2020; Ilany and Akçay 2016; Firth et al. 2017). Birth and death rates can also shape social network structure by altering the selective advantage of different social behaviours. For example, if the initial costs of forming a social relationship are high or the benefits are gained gradually over time, then we might expect such relationships to occur more often in species with longer lifespans (Silk and Hodgson 2021). As a species' position on the fast‐slow continuum is closely linked to generation time, it might be expected to influence social network structure and dynamics (Silk and Hodgson 2021). However, the extent to which variation in social network structure across the fast‐slow continuum can be explained as a direct outcome of demography remains unknown (Silk and Hodgson 2021).

Despite clear links between demography and social structure, demographic processes are rarely incorporated in social network models (Shizuka and Johnson 2020; Pellis et al. 2015), with networks typically either static in size and structure (Ferreri et al. 2014; Gallos and Fefferman 2015), or being dynamic but with constant size (Li et al. 2011; Fefferman and Ng 2007b). This restricts the relevance of such studies over longer timescales, where demographic influence on network structure cannot be neglected (Shizuka and Johnson 2020). Theoretical network modelling approaches incorporating more realistic demographic dynamics could be effective tools to better understand how social structure varies across the fast‐slow continuum.

Social network structure and dynamics can also be linked to individual‐level social preferences (Croft et al. 2016). Diverse preferential behaviours are observed in nature, including for social relationships based on particular traits, such as larger physical size in greater kudu ( *Tragelaphus strepsiceros* ) (Owen‐Smith 1993) or greater maturity in African elephants (McComb et al. 2001) or phenotypic similarity (Massen and Koski 2014; Ebenau et al. 2019; McPherson et al. 2001; Croft et al. 2009). Therefore, models of social structure and dynamics should incorporate preferential behaviour (Brask et al. 2024). Such an implementation was introduced by Fefferman and Ng (2007b). Here, each individual was assigned a social rule and frequently reevaluated their social relationships with respect to this rule, altering their own network accordingly. Social rules were implemented in the form of network centrality measures, describing each individual's network position (Freeman 1978; Sosa et al. 2021), with different measures capturing a range of natural preferential behaviours (Fefferman and Ng 2007b). Their study showed that variation in these individual‐level social rules drove complex differences in emergent group‐level network structures. Their model has since been extended to address other ecological (Hock et al. 2010; Hock and Fefferman 2011; Greening and Fefferman 2014) and epidemiological (Hock and Fefferman 2012; Gallos and Fefferman 2015; Fefferman and Ng 2007a; Wilson et al. 2020) questions. However, the ecological and evolutionary conclusions drawn are limited since they do not account for demographic processes, which are likely to be important over longer timescales.

A fundamental consideration when integrating demographic replacement into dynamic social network models is *social inheritance*: if and how social relationships are passed from a parent to its offspring (Shizuka and Johnson 2020; Ilany and Akçay 2016; Ilany et al. 2021). One model of social inheritance (Ilany and Akçay 2016) suggested that network stability could be achieved through individuals socialising with the social circle of their parents, and this model successfully replicated aspects of the observed social network structure of four mammal species. In contrast, parent and offspring social relationships may be disjoint, such as when species exhibit abandonment behaviour (Davenport et al. 2019). Therefore, different forms or extents of social inheritance may also shape long‐term animal social dynamics. Recent studies on inheritance patterns in spotted hyenas ( *Crocuta crocuta* ) (Ilany et al. 2021) and wild baboons (*Papio* spp.) (Roatti et al. 2023) highlight how particular forms of inheritance can have long‐lasting influences upon individual social relationships. The role of social inheritance may be especially significant for fast‐lived species, if network dynamics are strongly governed by the more rapid replacement of individuals (Ilany and Akçay 2016; Shizuka and Johnson 2020). It is therefore important to consider social inheritance when assessing how demography may constrain the emergence of social network structures (Ilany and Akçay 2016; Shizuka and Johnson 2020).

Here we extend the dynamic social network model of Fefferman and Ng (2007b) to include demographic replacement and social inheritance. We consider popularity‐ and closeness‐based social rules (as in Fefferman and Ng 2007b), alongside homophily‐based rules, which broaden the range of behaviours captured. While our model (or *any* model) cannot capture the full diversity of social organisation across the animal kingdom, its clear assumptions allow conceptualisation of the potential for demographic constraints to influence emergent social network structure and the generation of new hypotheses that can be tested with empirical data. We assess whether demographic constraints differ across the fast‐slow continuum, modes of social inheritance and social rules. Our key finding is that speed of life can constrain social network structure, determining the extent to which particular social rules shape social organisation. For species with longer generation times, emergent properties of the network structure were primarily influenced by social preferences, while for shorter generation times social inheritance became more important. This relationship between social structure and generation time caused by demographic constraints could then ultimately drive the co‐evolution of life history and sociality.

## 2 Methods

### 2.1 Initialisation

The social network of a population was modelled as a directed graph $G=\left(V,E\right)$ where V is the set of nodes, each representing an individual, and E is the set of directed edges representing (not necessarily mutual) preferences for social relationships between individuals (social inclinations that underlie patterns of affiliative behaviour, social support or association; Hinde 1976; De Moor et al. 2024). The graph was initialised with N=100 nodes, each having five outward edges assigned from a uniform distribution across the population. Duplicated edges were not allowed, nor edges which connected a node to itself. Additionally, in order to consider homophilous social dynamics, all nodes were assigned a phenotype (‘trait’) value, T, taken from a truncated normal distribution, $N\left(0.5,0.1\right)$, restricted to the interval $\left[0,1\right]$.

### 2.2 Demographic Replacement

The demographic process was modelled as a continuous‐time discrete‐space Markov chain, which was sampled using the Doob‐Gillespie Algorithm (Doob 1942; Gillespie 1977). Here, individuals were assigned a birth rate b−qN, and death rate d, where b is the maximum birth rate, N is the current total population and $q=\left(b-d\right)/K$ is the population's susceptibility to crowding, with K denoting the population carrying capacity. For a given demographic event, each individual thereby had a probability of giving birth, $P_{\text{birth}}$, and a probability of death, $P_{\text{death}}$, corresponding to an increase or decrease in the population size, respectively (see Equations (1), (2)).

(1) $$P_{\text{birth}}\left(N\right)=\frac{b-\mathrm{qN}}{R\left(N\right)},\;\left(N\to N+1\right)$$

(2) $$P_{\text{death}}\left(N\right)=\frac{d}{R\left(N\right)},\;\left(N\to N-1\right)$$

Here, $R\left(N\right)=N\left(b-\mathrm{qN}\right)+\mathrm{dN}$ is the sum of the transition rates of the Markov chain at the current state N. The time between demographic events was drawn from an exponential distribution with parameter $\lambda =R\left(N\right)$. Note, if $P_{\text{birth}}<0$ it was set to zero. Note that the average population dynamics were logistic (Caswell 2006), and that by using a Markov chain model we have assumed that birth and death processes for each individual are independent. The demographic processes included density dependence to ensure population resilience around the carrying capacity K, and because density‐based effects are important for population‐level predictions (Hernández et al. 2026). We assumed that density dependence acts only on birth rates, as this reduces the size of the population fluctuations (as shown in Supporting Information Section S1.1).

We captured position on the fast‐slow continuum by scaling the birth and death parameters. We first defined the ‘fastest’ demographic parameters (representing the shortest generation time) as $\left(b_{f},d_{f}\right)=\left(10,1\right)$. Then, utilising a scaling by $c\in \left[0,1\right]$, we used these fastest parameters to obtain a slower speed of life $\left(b,d\right)=\left({\mathrm{cb}}_{f},{\mathrm{cd}}_{f}\right)$. A higher value of c therefore corresponds to a faster speed of life. We also scaled q by c to maintain the carrying capacity K=100. For mathematical details on how c correlates with speed of life see Section S1.2, and regarding the choice of values for $b_{f}$ and $d_{f}$ see Section S1.3. We note that both generation time and time until first reproduction, established empirical and theoretical measures of position along the fast‐slow continuum (Gaillard et al. 2005), vary *inversely* with c (Section S1.4).

### 2.3 Social Inheritance

To integrate demographic processes into the network structure, our model included *social inheritance*, here referring to (nongenetic) inheritance of social relationships from parent to offspring, and excluding inheritance of any other social/behavioural trait. Upon the birth of a new individual, we considered three different modes of social inheritance: (i) *Zero*, where no edges were assigned to the newborn at birth, (ii) *Full*, where newborns were assigned the same outward edges as their parent and (iii) *Parental*, where newborns were given an outward edge to their parent only. Whenever an individual died, their corresponding node was removed from the graph along with all their edges.

We also included inheritance of trait values. The new node (individual) was assigned the trait value $T_{o}∼N\left(T_{p},m\right)$, where $T_{p}$ is the trait value of the parent and m=0.25 is the mutation variance. Trait values were fixed to the range $\left[0,1\right]$ using reflective boundary conditions. The value m=0.25 was chosen so that the effects of genetic drift were negligible, ensuring the distribution of trait values did not narrow during simulations.

### 2.4 Social Preferences

We modelled the social dynamics (emerging from preferential behaviour) as periodically occurring *social updates*, following the methodology of Fefferman and Ng (2007b). Specifically, we assumed that individuals act selfishly to maximise the quality of their own relationships (edges) according to a social rule, which represents their social preference. Prior to the social update step, each node in the graph had between 0 and 5 outward edges. Nodes with greater than 3 edges could improve the quality of their relationships by dropping their worst scoring ones based on their social rule, until exactly 3 remain. Then, all nodes formed new outward edges to other nodes, chosen uniformly from V but excluding their current and recently removed edges, until each individual has 5 out edges. We assumed the frequency of social updates is independent of generation time.

During a social update, individuals were scored using one of the three individual‐level scores given in Table 1, each corresponding to one of the social rules considered: (i) Popularity, where a social relationship was preferred if it had a high in‐degree (high number of incoming edges), (ii) Closeness, where a social relationship was preferred if that individual had high closeness centrality, meaning it was well‐integrated socially with a strong ability to reach others within the network and (iii) Homophily, where a social relationship was preferred if it was with an individual of a more similar trait value. In each simulation, all individuals followed the same social rule.

**TABLE 1:** The individual‐level and network‐level measures considered in this study. Notation: I V is the set of nodes (individuals), E is the set of edges, $T\left(i\right)$ is the trait value of individual i, $\deg_{\text{in}}\left(i\right)$ is the in‐degree of individual i (meaning the number of edges going *into* this individual), N=∣V∣ is the total number of individuals, $P_{\max}$ is the maximum individual‐level popularity score in the network and $v\left(i,j\right)$ is the length of the shortest path from individual i to individual j (where $v\left(i,j\right)=N$ if no path exists). The popularity and closeness measures here are as defined in Fefferman and Ng (2007b). Individual measures drive social updates and global measures evaluate organisational success and describe structural properties of the network.

Measure	Formula	Description
*Individual‐level scores*		
Popularity	$P\left(i\right)=\frac{\deg_{\text{in}}\left(i\right)}{N-1}$	Measures the proportion of other individuals in the population choosing to affiliate with a given individual.
Closeness	$C\left(i\right)=\sum_{j\neq i}\frac{N-1}{v\left(i,j\right)}$	Average path length from one individual to all others in the network relative to network size.
Trait Difference	$D\left(i,j\right)=\left|T\left(i\right)-T\left(j\right)\right|$	Measures the dissimilarity of a pair of individuals, using the Euclidean distance between their trait values
*Network‐level measures*		
Popularity	$P\left(G\right)=\sum_{i\in V}\frac{\left(P_{\max}-P\left(i\right)\right)}{\left(N-2\right)}$	Measures the average divergence from the most popular individual, and therefore how centralised the network is.
Closeness	$C\left(G\right)=\sum_{i\in V}\frac{C\left(i\right)}{\left(N-1\right)\left(N-2\right)}$	Average closeness score across all nodes in the network, and therefore the level of network cohesiveness.
Average Trait Difference	$D\left(G\right)=1-\frac{1}{|E|}\sum_{\left(i,j\right)\in E}D\left(i,j\right)$	Average trait difference across all edges in the network, scaled so a higher score means greater average assortativity or homophily.

### 2.5 Model Overview and Simulation

**FIGURE 1:**
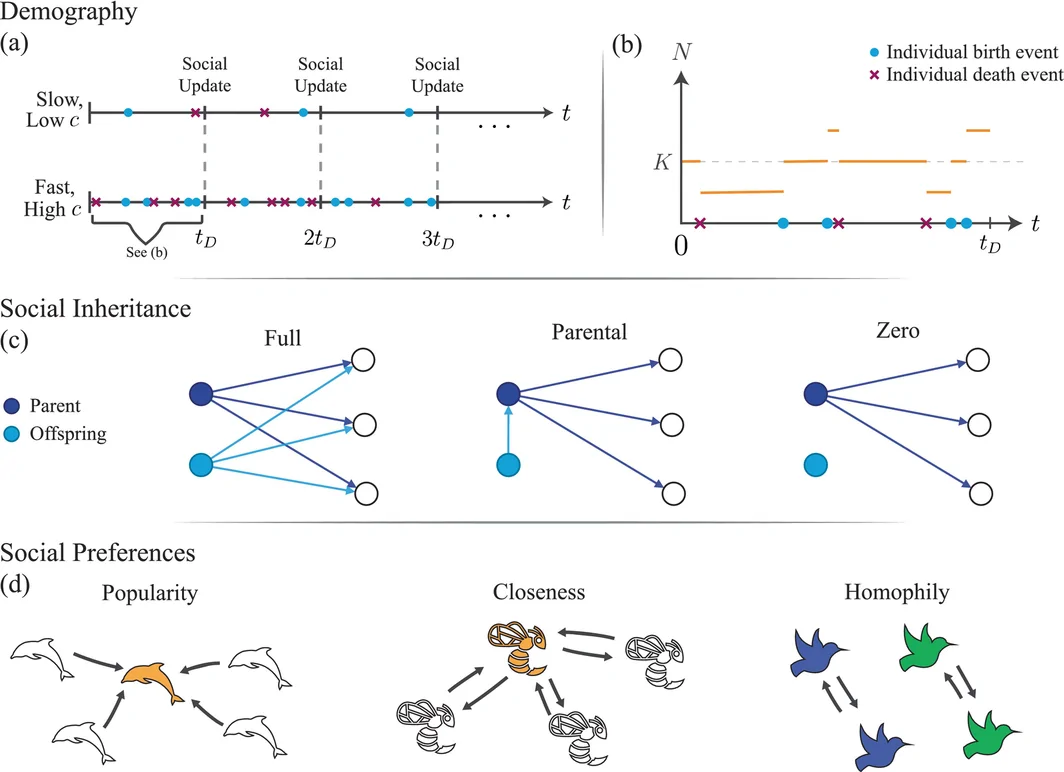
Graphical representation of the model components. (a) Timeline of the model, highlighting the position of the social updates alongside birth (blue dots) and death (purple crosses) events, with (top) long (bottom) short generation times. (b) Example of the changes in population size in one period between social updates, as a realisation of a continuous‐time Markov Chain model. (c) The different social inheritance schemes, with a parent (dark blue), individuals they are connected to (white) and their offspring (light blue) alongside the relationships assigned at birth. (d) The different social rules. For popularity and closeness individuals aim to connect to high scoring individuals (orange). For homophily individuals of similar traits aim to connect with each other (dark blue with dark blue, and green with green).

After initialisation, demographic processes were run for a period of $t=t_{D}$. Within this process if a new individual was born, we implemented a mode of social inheritance (either full, parental or zero). If a death occurred, all edges for that individual were removed. After the time $t=t_{D}$ elapsed, a ‘social update’ was implemented. Then the demographic processes continue for another duration of $t=t_{D}$. This cycle was repeated for a sufficient period of time for the emergent structural properties of the social network to converge to a quasi‐stable state (500 social updates). Each component of the full model is visualised in Figure 1. We chose $t_{D}=1$ without loss of generality (see Section S1.6). Note, when c=0 our model is identical to that of Fefferman and Ng (2007b), which therefore functioned as null model to our own.

We quantified differences in social structure with varying generation time by recording network‐level measures (Table 1) after each social update. These measures indicate the extent of social organisation of the group or population according to one of three social rules: popularity, closeness and homophily. Across the fast‐slow continuum (100 values of c), we performed 100 simulations for each social inheritance mode and social rule in order to analyse how the network‐level scores changed with time. The recorded network‐level measures were then taken as the mean (over the 100 simulations) of the final 100 social updates. Further simulation details are provided in Section S1.5.

## 3 Results

Our simulations examined how speed of life, social inheritance and social rules/preferences collectively influence emergent social network structure. Changes in generation time were found to impact dynamic social network structure, with the strength of this impact dependent upon both social inheritance and the social rules used by individuals to update their local networks. When generation times were long, emergent social network structures were primarily determined by the form of social preferences, whereas when generation times were short, social inheritance played a larger role, because demographic replacement is typically more rapid in fast‐lived species, so there were fewer opportunities for preferential social updating in the average lifespan.

### 3.1 Popularity‐Driven Social Rules

**FIGURE 2:**
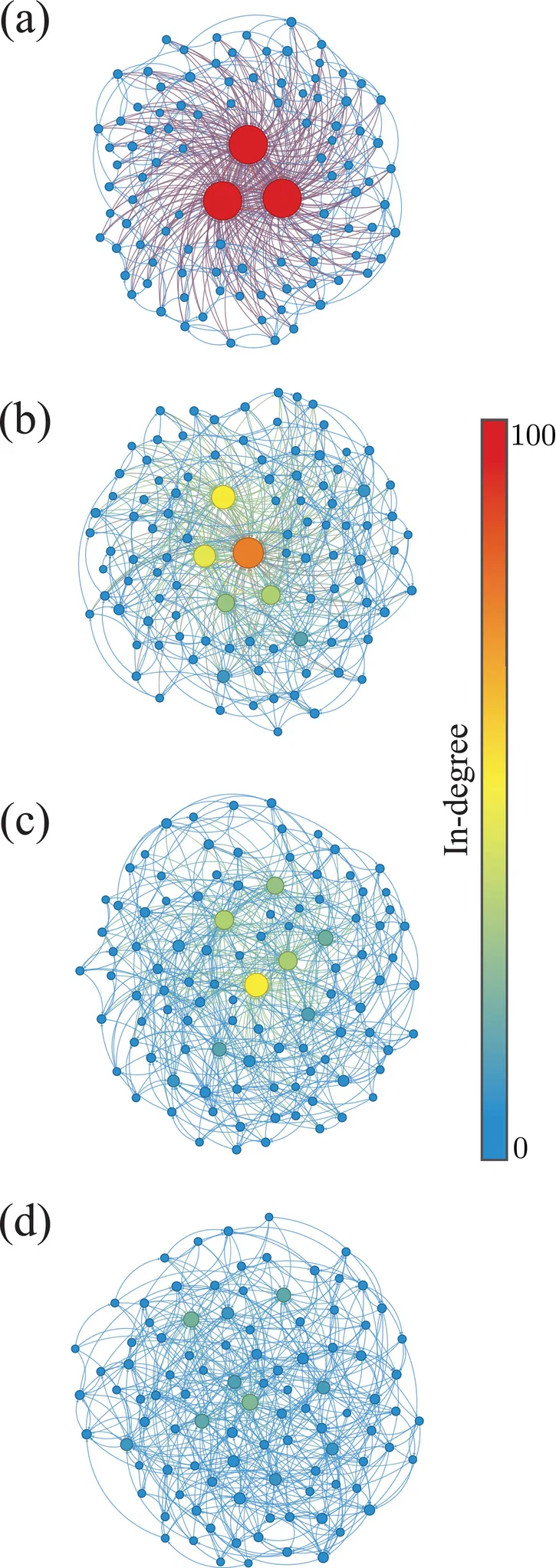
Example social networks obtained as a final output of the model for a group with popularity‐driven social preferences and decreasing generation time, full social inheritance and demographic replacement rate scaling (a) c=0, (b) c=0.01, (c), c=0.1 and (d) c=0.2. Nodes are sized and coloured according to their in‐degree.

**FIGURE 3:**
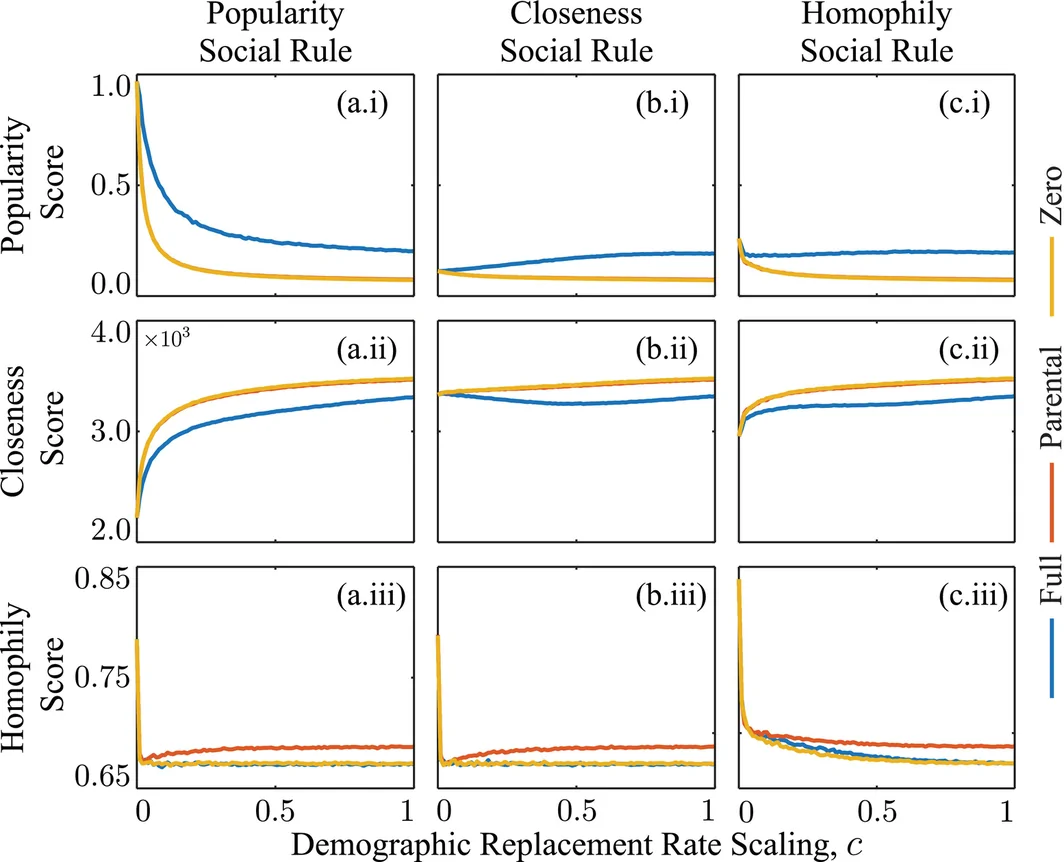
Variation of the average network‐level measures with the rate of demographic replacement, represented through variation in c. Social rules are: (a) popularity, (b) closeness and (c) trait. Network‐level scores are: (i) popularity, (ii) closeness and (iii) homophily. Social inheritance modes are: Full (blue), parental (red) and zero (yellow). Whenever the parental (red) curve does not appear in a subplot, it had a negligible difference from the zero (yellow) curve.

When individual social rules were based on the popularity measure, there was substantial variation in network structure across the fast‐slow continuum. In our baseline model, with the absence of demographic replacement (c=0), a highly centralised social network structure with three ‘central hubs’ (Figure 2a) was observed, as in Fefferman and Ng (2007b). However, with a higher rate of demographic replacement (i.e., shorter generation time) the networks became less centralised (Figure 2), but more cohesive, quantified by the rapid decay in network‐level popularity scores and increasing (then saturating) network‐level closeness scores as the rate of demographic replacement increases (Figure 3a i,ii). Such variation was due to the deaths of central hubs, which caused simulated networks to get stuck in non‐stable states while attempting to converge towards the structure shown in Figure 2a. Higher mortality led to more frequent deaths of central hubs, which resulted in networks being stuck in an earlier phase of convergence on average, where centralisation was lower. Trait assortativity scores of the emergent networks showed low variation with generation time, except in the absence of demography (c=0), where the score was anomalously dependent upon initial conditions (Figure 3a iii).

The mode of social inheritance affected the strength of the relationship between generation time and social network scores by mitigating the impact of the central hub removal upon network centralisation. With full social inheritance, the magnitude of changes in the structural properties of the network was smaller than with zero or parental social inheritance as full social inheritance led to more rapid re‐emergence of central hubs. For zero and parental social inheritance, average network‐level popularity and closeness scores were the same. However, parental social inheritance resulted in slightly higher levels of assortativity by trait value, since offspring had a similar trait value to their parent with whom they were initially connected (Figure 3a iii).

### 3.2 Closeness‐Driven Social Rules

When individual social rules were based on closeness, changes in the rate of demographic replacement caused only small changes in the properties of the network structure (Figure 3b). This invariance of the network structure across the fast‐slow continuum might have been due to the sensitivity of the closeness measure, as rewiring a single edge could dramatically change a node's closeness score, which increases the difficulty of searching for high‐scoring edges. Therefore, social dynamics were generally less influential under a closeness social preference (Hock et al. 2010; Fefferman and Ng 2007b) so were less affected by demographic replacement. As with popularity‐driven social dynamics, full social inheritance led to higher centralisation and lower cohesiveness than other inheritance modes, and parental social inheritance led to slightly higher trait assortativity. Network centralisation slightly increased with the speed of life history, but only with full social inheritance. This occurred because full social inheritance super‐additively increased the popularity of keystone individuals; being connected to more potential parents made them more likely to gain additional edges through inheritance, resulting in higher centralisation. The tendency for full social inheritance to lead to higher popularity scores, most prominent at faster replacement rates, was consistently observed across all social rules considered (Figures 3a–ci and 4d i).

**FIGURE 4:**
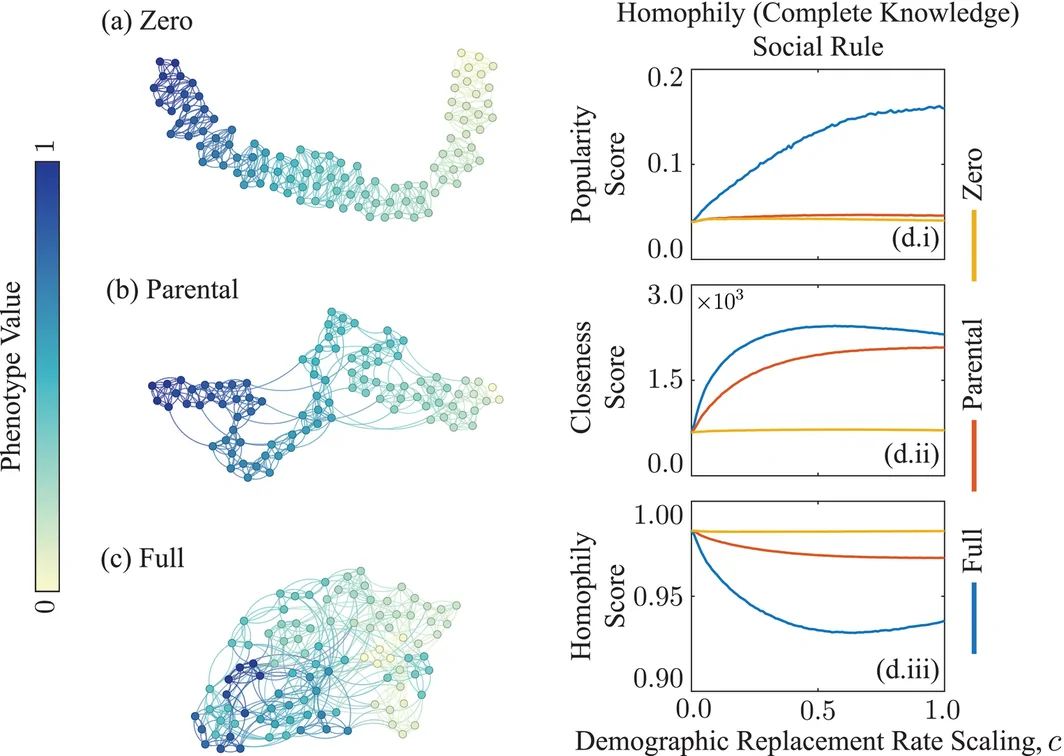
Model results with trait‐based social rules with the complete knowledge model modification. (Left) Example social networks obtained from the model with replacement rate scaling c=0.5 and social inheritance modes: (a) zero, (b) parental and (c) full. Darker colours correspond to higher trait values. (Right) The variation of the network‐level measures with the replacement rate scaling, c. Network‐level scores are: (i) popularity, (ii) closeness and (iii) homophily. Social inheritance modes considered are full (blue), parental (red) and zero (yellow).

### 3.3 Trait‐Driven Social Rules

When social rules were based on the trait‐homophily measure, the effect of variation in demographic replacement rate on the network properties was limited (Figure 3c). As in popularity‐driven networks, the change in network‐level popularity and closeness scores with increasing demographic replacement was initially rapid, but then saturated. However, the magnitude of the decline was smaller, and there were smaller differences between the network structures of fast‐lived and slow‐lived species when compared to results with the popularity social rule. The key difference observed under trait‐based social rules was a slight increase in network assortativity at longer generation times when compared to other social rules (Figure 3c iii). Nevertheless, this increase in assortativity score rapidly declined as speed of life increased (see Section S2.1).

### 3.4 Trait‐Driven Social Rules (Complete Knowledge)

For the homophily‐driven social preference, we additionally considered the scenario where connections added during a social update were not chosen at random but were instead to the most preferred individuals (those with the most similar trait value). We call this additional scenario *complete knowledge*, representing the case where social preferences were based on an easily observed and assessed phenotype. With complete knowledge, the resulting network dynamics and emergent structure were dramatically different to all other schemes considered and, in some circumstances, the emergent social structure was highly organised (Figure 4a–c). Popularity and closeness scores were lower than any previously tested scenario (compare Figure 4d i,ii with Figures 3a–c i and 3a–c ii). However, complete knowledge led to much higher assortativity scores (compare Figure 4d iii to Figure 3a–c iii) across the fast‐slow continuum and all social inheritance modes. Overall, complete social knowledge led to social dynamics which were more robust to the impact of demographic replacement.

Increasing the rate of demographic replacement did still impact network dynamics, by increasing the proportion of social relationships formed through inheritance, evidenced by network properties being independent of position on the fast‐slow continuum with zero social inheritance (Figure 4d i–iii). With complete knowledge, social dynamics only add connections to individuals with the most similar trait value, whereas inherited connections may have less similar trait values due to trait mutations. Therefore, with zero social inheritance there was always a maximally assortative network structure after a social update. Such structures (shown in Figure 4a) had low centralisation and cohesiveness. In contrast, with full social inheritance, as the rate of replacement increases, more edges are obtained through social inheritance (and these are less strongly assortative). As such, network assortativity decreased while popularity and closeness scores increased (Figure 4c). Parental inheritance balanced network assortativity and cohesiveness; parent‐offspring edges formed “bridges” across the network between individuals with potentially different trait values, increasing closeness but without sacrificing assortativity, due to the sparsity of these less assortative social relationships (Figure 4b). Note that this effect depends on trait heritability; reducing m (increasing heritability) would cause results for parental and full social inheritance to be closer to those for zero social inheritance.

## 4 Discussion

Our study linked generation time with social structure, highlighting how variation in life history strategies may help explain variation in social network structures. Our key finding was that more pronounced patterns of self‐organisation were a more common feature of animals with slow life history. Slower‐lived groups exhibited greater capacity for effective self‐organisation (aligning with previous work in primates; Demetrius et al. 2024), while faster‐lived groups exhibited networks which struggled to self‐organise due to demographic constraints. Hence, social systems of faster‐lived species may be more dissipative, or farther from their desired network structure (‘equilibrium state’) (Green et al. 2008). Furthermore, we showed that the emergent social network structure was primarily determined by social preferences for slow‐lived species and by the mode of social inheritance for fast‐lived species. Our homophily social rule highlighted the importance of social knowledge in determining the strength of social organisation across the fast‐slow continuum. Overall, our study explicitly linked generation time with the social structure of natural systems, highlighting how variation in generation time, hence in life history strategies, may help to explain the variation in social network structures observed in nature.

### 4.1 Impact of Social Inheritance Across the Fast‐Slow Continuum

We found that social inheritance influenced emergent social structure, with some patterns consistent across different social rules. A higher level of social inheritance produced networks with higher popularity scores and lower closeness scores for all considered social rules without complete knowledge. Hence, greater social inheritance can consistently result in more centralised and less cohesive social structures, with consequences for other ecological processes. For example, centralised networks may result in possible ‘superspreader’ events, where outbreaks of a simple contagion (such as an infectious disease) may be less likely but more explosive when they occur (Lloyd‐Smith et al. 2005). Although *dynamic* social organisation may restrict the impact of these individuals (Hock and Fefferman 2012). Contrastingly, more cohesive social networks have shorter average path lengths between individuals, so they may spread a complex contagion (such as novel behaviours) more effectively (Firth 2020), and simple contagions may also spread more effectively (although less explosively).

Social inheritance might also influence the relationship between generation time and the properties of the social network. For example, when social rules were defined by the closeness measure, the mode of social inheritance determined whether some network‐level scores were increasing or decreasing with speed of life. Without investigating multiple social inheritance modes, this nuanced impact of demographic replacement may not have been observed. Therefore, inheritance of relationships might play a key role in determining how social dynamics shape network structure, suggesting that network modelling studies should explicitly consider various forms of social inheritance whenever demographic processes are included (Ilany and Akçay 2016).

### 4.2 Role of Knowledge in Social Network Dynamics

Greater social knowledge (of the group as a whole–‘complete knowledge’–rather than an individual's knowledge of its own social network) was found to reduce the demographic constraint upon social organisation. Without complete knowledge, we found that replacement restricted assortativity; highly structured networks only emerged at long generation times. Therefore, we expect homophily by less visible traits, such as personality or social status, to be more common with slow‐lived species. With complete knowledge, emergent network structure was highly assortative across the entire fast‐slow continuum, represented by consistently higher homophily scores. We hence predict that we might observe strong homophily in social relationships by easily observed phenotypes, like colour, across more natural systems, as assortativity could be more successfully obtained through self‐organisation across the entire fast‐slow continuum. While both these kinds of homophily occur in nature (physical homophily: Mourier et al. 2012; Kelley and Evans 2018, personality homophily: Bhattacharjee et al. 2024; Massen and Koski 2014; Ebenau et al. 2019; Silk et al. 2003; Seyfarth et al. 2012; McPherson et al. 2001), their relative contributions to social structure across different ecological contexts remain an open question, and our results provide some theoretical predictions to guide empirical work.

When individuals had complete knowledge, greater levels of social inheritance led to more cohesive network structures, but reduced assortativity. Parental inheritance yielded a high cohesiveness with a smaller reduction in assortativity, representing a strong balance of high network cohesiveness and high assortativity. For fast‐lived species this balance was particularly effective (Figure 4d ii,iii). Comparatively, existing theory predicts that parental relationships are more likely in slow‐lived systems, due to the significant long‐term fitness advantage of parental care (Klug and Bonsall 2014). While we observed that lowering generation time greatly impacted network structure by increasing the abundance of socially inherited relationships, other ecological and evolutionary phenomena uncaptured in our self‐organisation framework have the potential to interact with these dynamics, further shaping social topology in some systems. Collectively, our results for trait‐based preferential behaviour highlight the potentially key role of social information in social network organisation (Gokcekus et al. 2021) and demonstrate how this might vary across the fast‐slow continuum.

### 4.3 Future Directions: Co‐Evolution of Social Behaviour and Speed of Life

Our results suggested that position on the fast‐slow continuum and social behaviour (represented here by the different social rules and modes of inheritance) jointly influence social network structure. Therefore, we speculate these features might co‐evolve to shape emergent network structure. Individual social network position has been related to fitness both directly (Brask et al. 2021; Snyder‐Mackler et al. 2020) and indirectly through correlation with fitness‐related traits (Pasquaretta et al. 2014; McDonald 2007; Silk et al. 2010; Ellis et al. 2017). Group network structure has also been shown to influence individual fitness (Ebensperger et al. 2016; Evans et al. 2021; Ballard and Robel 1974; Clarke and Faulkes 1997). Since social preferences influenced network properties most prominently at long generation times, selection pressures on social preferences might be stronger when the speed of life history is slow. This aligns with the hypothesis that for slower‐lived species (reciprocal) social behaviours are more evolutionarily viable, as their associated costs (including initial formation) are offset by the benefits which accumulate over the longer lifespan (Silk and Hodgson 2021). In contrast, for fast‐lived species, selection may be more likely to operate on social behaviours which are more directly related to demography (e.g., inheritance), because demographic processes influence emergent network properties more strongly. We also suggest that selection on generation time may vary between groups with different social behaviours. Many group‐living species benefit from having centralised structures featuring keystone individuals (Ballard and Robel 1974; Knörnschild et al. 2009; Clarke and Faulkes 1997). Our results suggest that this could lead to selection for a combination of longer generation times and popularity‐driven preferential behaviour (see Figure 3a i), as this drives the emergence of substantially higher network centralisation. Yet, keystone individuals who are critical to this centralised structure may have different individual fitness functions (Lloyd‐Smith et al. 2005; Aplin et al. 2012). Therefore, social behaviour may co‐evolve with generation time due to the collective influence of these features upon network structure uncovered in this study, with potential for multi‐level selection.

Our model considers social behaviour and life history as related through the influence of demography upon self‐organising social processes, allowing us to suggest an evolutionary relationship between these features. However, there are other ways in which these features may be linked. For example, life history may alter the selective advantage of different social behaviours (Silk and Hodgson 2021), influence metabolic rates (Auer et al. 2018), and potentially induce pace‐of‐life syndromes (Réale et al. 2010), influencing social behaviour and hence network structure. Considering these additional factors may be important to fully understand the evolutionary relationship between life history and social behaviour. We could extend the model to explicitly represent evolutionary processes. For example, we could generate new socio‐evolutionary theory by allowing individual birth and death transition probabilities (Equations (1) and (2)) to depend upon individual social position and network‐level structure.

### 4.4 Future Directions: Differentiated Social Relationships Across the Lifespan

In this study we modelled demographic variation through changes in birth and death rates, neglecting the role of senescence. In natural populations the quality and quantity of social relationships can vary across an individual lifespan (Siracusa et al. 2022; Wrzus et al. 2013). Such social ageing effects could be included in our model by implementing both weighted edges and variation in the number of social relationships which individuals aim to maintain. If older individuals maintain only their strongest social relationships (Sadoughi et al. 2024), then we may expect increased within‐group variation in social position. A weighted implementation of our model could provide new hypotheses on how social ageing influences emergent network properties across the fast‐slow continuum, and also provide the flexibility to model more diverse social behaviours. For example, slow‐lived species are hypothesised to have stronger kin‐based social relationships (Zipple et al. 2024), which may have evolutionary consequences if extended parent‐offspring overlap facilitates broad inter‐generational transfers (e.g., of care), which in turn influence life histories. We could also capture the effects of differentiated parent‐offspring social relationships upon emergent network structure, thereby potentially providing new insight into the evolution of care.

### 4.5 Future Directions: Within‐Group Heterogeneity

In our model, we assumed that the group/population had homogeneous social behaviour—individuals aimed to maintain the same number of out‐connections and to follow identical social rules. As in Fefferman and Ng 2007b, after a social update all individuals had an out‐degree of 5 and were willing to drop (at most) 2 relationships when adjusting their networks. We predict that increasing this ratio beyond 5:2 would lead to slower self‐organisation (making emergent structure more sensitive to changes in generation time) but also to more organised emergent structure when generation time is sufficiently long. It may be interesting to examine if variation in this ratio can lead to *connectivity criticalities*, where a large structural change arises from slight behavioural variation (Green et al. 2008). Differences or heterogeneities in the number of social relationships held by individuals or in the social rules they follow could easily be implemented in future work (Ng 2008; Brask et al. 2024), and this is an interesting direction given the potential importance of heterogeneity in ecological processes (Allan et al. 2025; Lloyd‐Smith et al. 2005; Stein 2011) and their interaction with social dynamics (Young et al. 2022, 2023; Hock and Fefferman 2011).

### 4.6 Model Limitations

Our model incorporates key characteristics of natural social system dynamics, but, like all models, it relies upon simplifying assumptions. First, we assume that species update their social relationships at the same rate, independent of lifespan, allowing the demographic turnover scaling parameter c to directly correlate with the speed of life history, facilitating interpretable model outcomes. However, we know little about how social updating varies across the fast‐slow continuum (Silk and Hodgson 2021), and pace of life syndromes or co‐evolutionary history may break this assumption. Faster‐lived species might update social relationships more frequently than slower‐lived species due to their greater metabolic rates (Réale et al. 2010) and bolder, more exploratory personalities (Silk and Hodgson 2021). In Section S2.2, we relaxed this assumption so the rate of social updates increases or decreases slightly with c and showed our results are qualitatively robust when the increase/decrease is small to moderate. It would be interesting to vary generation time and age at first reproduction independently (currently impossible as c inversely correlates with both these features), allowing examination of how specific aspects of demography influence social structure. Additionally, the (uniform) randomness of forming relationships makes it unlikely a simulated network contains multiple connected components. Therefore, our findings are most applicable for well‐mixed populations, such as species with fluid fission‐fusion dynamics (Aureli et al. 2008) or group‐living species such as eusocial insects (Waters and Fewell 2012), and are less applicable when we expect high social network modularity. Future work may consider different methodologies for forming relationships, perhaps giving higher weight to second‐order social relationships in the group, rather than equal weight between all group members.

## 5 Conclusion

We presented an initial examination of how demographic replacement might influence emergent patterns of social organisation, and how this relationship varies across the fast‐slow continuum. Our theoretical framework was novel in its inclusion of demography in a dynamic animal social network, and provides a foundational step towards understanding the role of social dynamics over long timescales, where demographic replacement cannot be neglected. We found that position on the fast‐slow continuum could be an important driver of emergent social network structure in natural populations. Particularly, the emergent network structure for slower‐lived species was most influenced by social preferences, while for faster‐lived species it was most influenced by social inheritance. We outlined how our key result sets up a suite of ecological and evolutionary predictions that could be verified by future meta‐analyses and empirical studies. Our model is flexible and modular, and can therefore be easily extended to address these general questions or to model behaviours of specific systems. Collectively, this work is an important first step towards understanding if and how the immense diversity in social structure and life histories observed in nature may be interwoven.

## Author Contributions

R.S.W., A.W., X.O. and M.J.S. conceived the study; R.S.W., A.W. and X.O. performed mathematical analysis; R.S.W. and X.O. wrote the simulation code; R.S.W. prepared the first draft of the manuscript; all authors contributed to the final version of the manuscript.

## Funding

This work was supported by Royal Society, URF/R1/221800. Biotechnology and Biological Sciences Research Council, BB/V00378X/1. Engineering and Physical Sciences Research Council, EP/S023291/1.

## Supporting information


**Figure S1:** Average population size with time from 10,000 stochastic simulations of the demographic model with density dependence upon birth and death. Solid line represents the average trajectory corresponding to Equation (B) and the shaded region represents one standard deviation from the mean trajectory. The parameters are *b* = 10, *d* = 1, *K* = 100, initial population size *N*_0_ = 25 and density dependence parameters *q*_*b*_, *q*_*d*_ determined by Equations (S.4) with (a) *l* = 0 (density dependence only upon death), (b) *l* = 0.03, (c) *l* = 0.06 and (d) *l* = 0.09 (density dependence only upon birth). Average trajectories have decreasing variance with increasing *l*.
**Figure S2:** Variation in the standard deviation of the population sizes (averaged across simulation time) with *l* across 10,000 stochastic realisations (per tested *l* value) of Equation (B). The parameters are *b* = 10, *d* = 1, *K* = 100, initial population size *N*_0_ = *K* and density dependence parameters *q*_*b*_, *q*_*d*_ determined by *l* according to Equations (S.4). Simulations run for period between *t* = 0 and *t* = 5.
**Table S1:** Transitions and their respective total rates for the stochastic birth‐death simulations, with the fastest and scaled demographic processes considered. Note, the transition rate for birth events is set to zero if *b*_*f*_ − *q*_*f*_ *N* < 0, for avoidance of negative transition rates.
**Table S2:** Parameter values and their brief descriptions. See main text for full definitions of parameters.
**Figure S3:** (a) Change in the network‐level score for one model realisation with the full‐inheritance strategy, popularity social preferences and demographic replacement rate scaling *c* = 0.0303 (black), and the mean of the final 100 iterations (red). (b) Final mean values of the 10,000 simulations for this particular pair of social inheritance and preference (light blue crosses) alongside the mean curve for each *c* value (dark blue). The particular simulation from the left is highlighted (red, slightly larger cross).
**Table S3:** Transition rates in the stochastic birth‐death simulation for both *t*_*D*_‐scaled and *c*‐scaled parameter choices. The transition rate for birth events is set to zero if *b*_*f*_ − *q*_*f*_ *N* < 0, for avoidance of negative transition rates. Note that we are grouping together all birth and death events for all individuals in the network, as to only consider the population‐level dynamics.
**Figure S4:** Examples of demographic processes, given as stochastic realisations of the stochastic birth‐death model (initialised at the carrying capacity) using the Doob‐Gillespie algorithm (Gillespie 1977; Gillespie 2007; Doob 1942; Doob 1945). Fixed parameters are $b_{f}=10$, $d_{f}=1$ and K=100. Different parameters are (top) c=1, $t_{d}=1$ and (bottom) c=0.5, $t_{d}=2$. The top figure shows more rapid population fluctuations than the bottom figure, but in a shorter time period.
**Figure S5:** The impact of trait‐based social rules with incomplete social knowledge upon the level of trait homophily of the network, quantified by the values of (a) $\Delta D_{p}$ and (b) $\Delta D_{C}$ for *c* ∈ [0, 1]. Social inheritance strategies considered are *full* (blue), *parental* (red) and *zero* (yellow).
**Figure S6:** Popularity scores with varying rates of demographic replacement, *c*, and for different relations between *c* and rate of sociality, using the relationship outlined in Section S.2.2. (Left) Different functional relations between speed of life and rate of sociality. (Right) The corresponding change in network‐level popularity scores with speed of life. Parameters used are as detailed in Table S2. The mode of social inheritance is zero‐inheritance.

## Data Availability

The simulation code underlying this study is publicly available at: https://github.com/rswalker‐eco/Fast‐Slow‐Nets. The simulated data used to generate the figures are publicly available at: https://doi.org/10.5281/zenodo.21250752.
